# Coronal Balance vs. Sagittal Profile in Adolescent Idiopathic Scoliosis, Are They Correlated?

**DOI:** 10.3389/fped.2019.00523

**Published:** 2020-01-10

**Authors:** Qichao Ma, Lin Wang, Lihua Zhao, Yicheng Wang, Mengjie Chen, Sun Wang, Zhibao Lv, Yi Luo

**Affiliations:** ^1^Department of Orthopedics, Shanghai Children's Hospital, Shanghai Jiao Tong University, Shanghai, China; ^2^Department of General Surgery, Shanghai Children's Hospital, Shanghai Jiao Tong University, Shanghai, China

**Keywords:** scoliosis, coronal balance, sagittal balance, pelvic profile, canonical correlation

## Abstract

**Background:** Clinical evaluation of the postural balance in adolescent idiopathic scoliosis has been evaluated by coronal and sagittal parameters. The relationship between coronal and sagittal parameters has not been fully studied in adolescent idiopathic scoliosis.

**Objective:** This study aimed to evaluate the relationship between coronal and sagittal parameters in idiopathic scoliosis.

**Methods:** One hundred and three patients of Adolescent Idiopathic Scoliosis (AIS) were recruited. Radiographs were evaluated for the following spine and pelvic measurements. Coronal parameters including thoracic Cobb angle (TC), lumbar Cobb angle (LC), global coronal balance (GCB), apical translation of the major curve (AT), and coronal pelvic tilt (CPT) were analyzed. Sagittal parameters including thoracic kyphosis (TK), lumbar lordosis (LL), sagittal vertical axis (SVA), pelvic incidence (PI), pelvic tilt (PT), and sacral slope (SS) were analyzed. A canonical correlation analysis was performed between all of the coronal parameters and sagittal parameters.

**Results:** One hundred and three patients were recruited (male 15, female 88; aged 12.5 ± 1.5 years). Compared with the published age-matched normal population, TK, PI, LL, and SS were not statistically different in the normal group. However, PT was significantly increased in patent groups (*P* = 0.003). Canonical correlation analysis showed strong correlation between coronal parameters and sagittal parameters, *F*_(20, 313)_ = 2.44, *p* = 0.001. SVA was not correlated with any coronal parameter.

**Conclusions:** Children with AIS showed an increased pelvic tilt sign. In this study, TC, LC, GCB, AT, CPT, TK, LL, SVA, PI, PT, and SS contributed to the postural balance. In AIS, coronal balance is contributed to sagittal balance.

## Introduction

Adolescent idiopathic scoliosis (AIS) is a three-plane deformity of the spine. Spinal deformities in AIS include changes in coronal and sagittal planes as well as axial rotation of the vertebrae and pelvis. The Cobb angle is still the golden standard to measure the magnitude of curves (1, 2). It can be used not only in coronal plane but also in sagittal plane to acquire parameters of lumber lordosis (LL) and thoracic kyphosis (TK). In terms of classifications, Lenke's scheme taken the place of King's classification has been adopted by the most spine surgeon (3).

Spine surgeons usually seek to correct postural imbalances in coronal and sagittal planes. Many postural parameters are related to poor functional balance in AIS (4). The balance control was also related to the location and severity of the curve (5, 6). Sagittal vertical axis (SVA), coronal balance (CB), and apical translation of the major curve (AT) are most common variables routinely used for postural balance assessment before and after spine correction (7–9). Sagittal pelvic parameters including pelvic incidence (PI), pelvic tilt (PT), and sacral slope (SS) are universally accepted. Literature has been reported that spinopelvic parameters were correlated to health-related quality of life in adult (10).

The relationship between the coronal and sagittal variables has not been evaluated up to date. The objective of this study is to investigate associations between the coronal and sagittal postural variables in AIS. A better understanding of these correlations will help spine surgeons improve surgical planning such as the choice of fusion levels and prediction of post-operative complications.

## Materials and Methods

### Patients' Clinical Information

A total of 103 teenagers with idiopathic scoliosis were recruited from spine clinic. Inclusion criteria included age over 10 years, normal ambulatory status, no leg length discrepancy, Cobb angle of >10 degrees, and no previous history of spine or lower extremity surgery.

### Radiographic Assessment

Postero-anterior and lateral spine radiographs on large 36 × 14 inch cassettes were taken for the recruited patients in the standing posture. Two senior spine surgeons carried out all the measurements in PACS systems. The average values were defined as the actual values.

**Figure 1:**
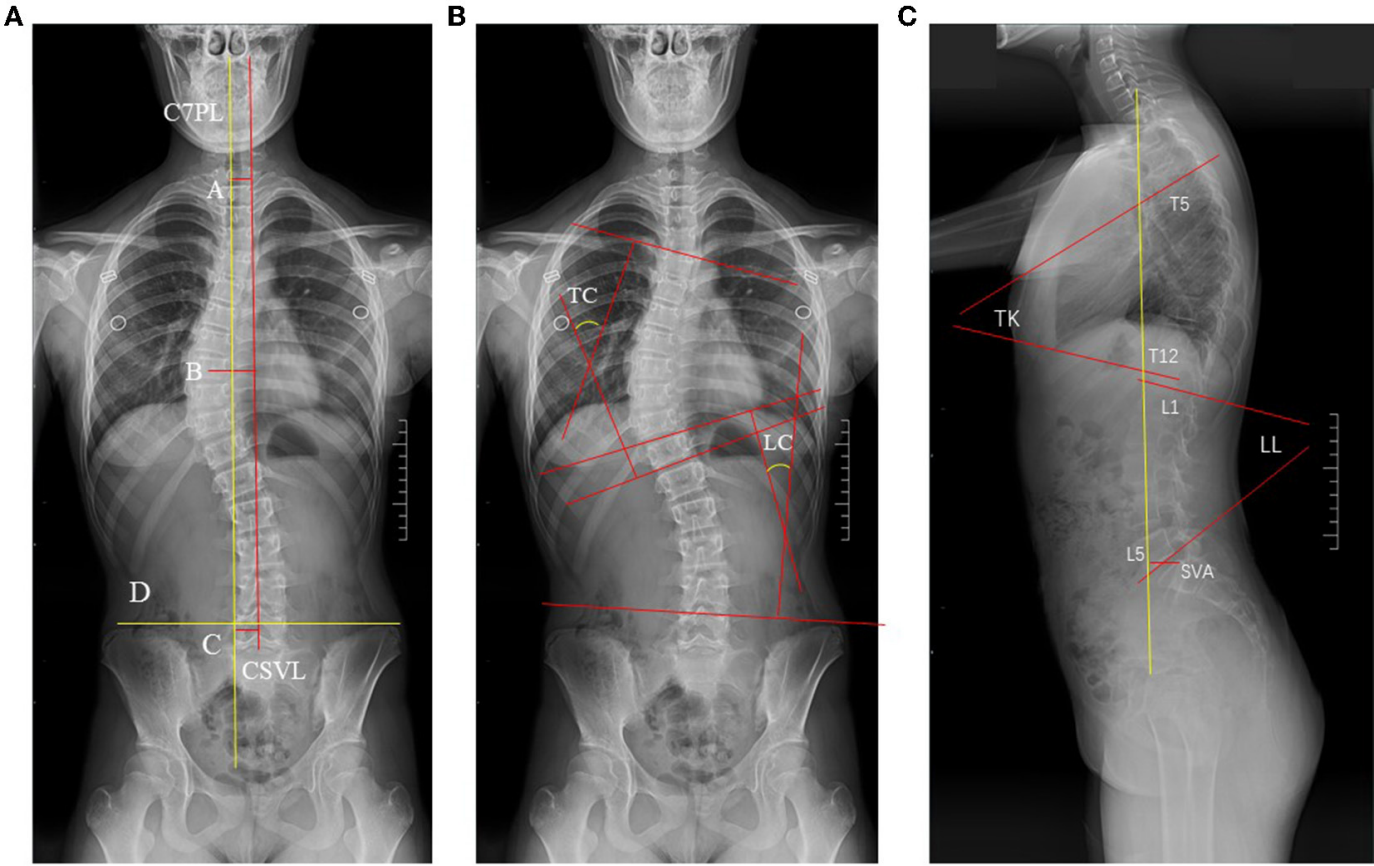
The postural balance parameters of C7PL, GCB, AT, CPT, CSVL, TC, LC, TK, LL, SVA. In **(A)**, the assessment of coronal balance. Line A (GCB) stood for the width between the central sacral vertical line (Line C, CSVL) and the mid-C7 vertebra plumb line. Line B stood for Apical translation of the major curve (AT) is measured as the offset between the center of the apical vertebra of the major curve and CSVL. Line D connected the highest point of the iliac wing on two sides, and CPT was the intersection of this line and the horizontal line. **(B,C)** Showed the parameters of TC, LC, TK, LL, SVA.

**Figure 2:**
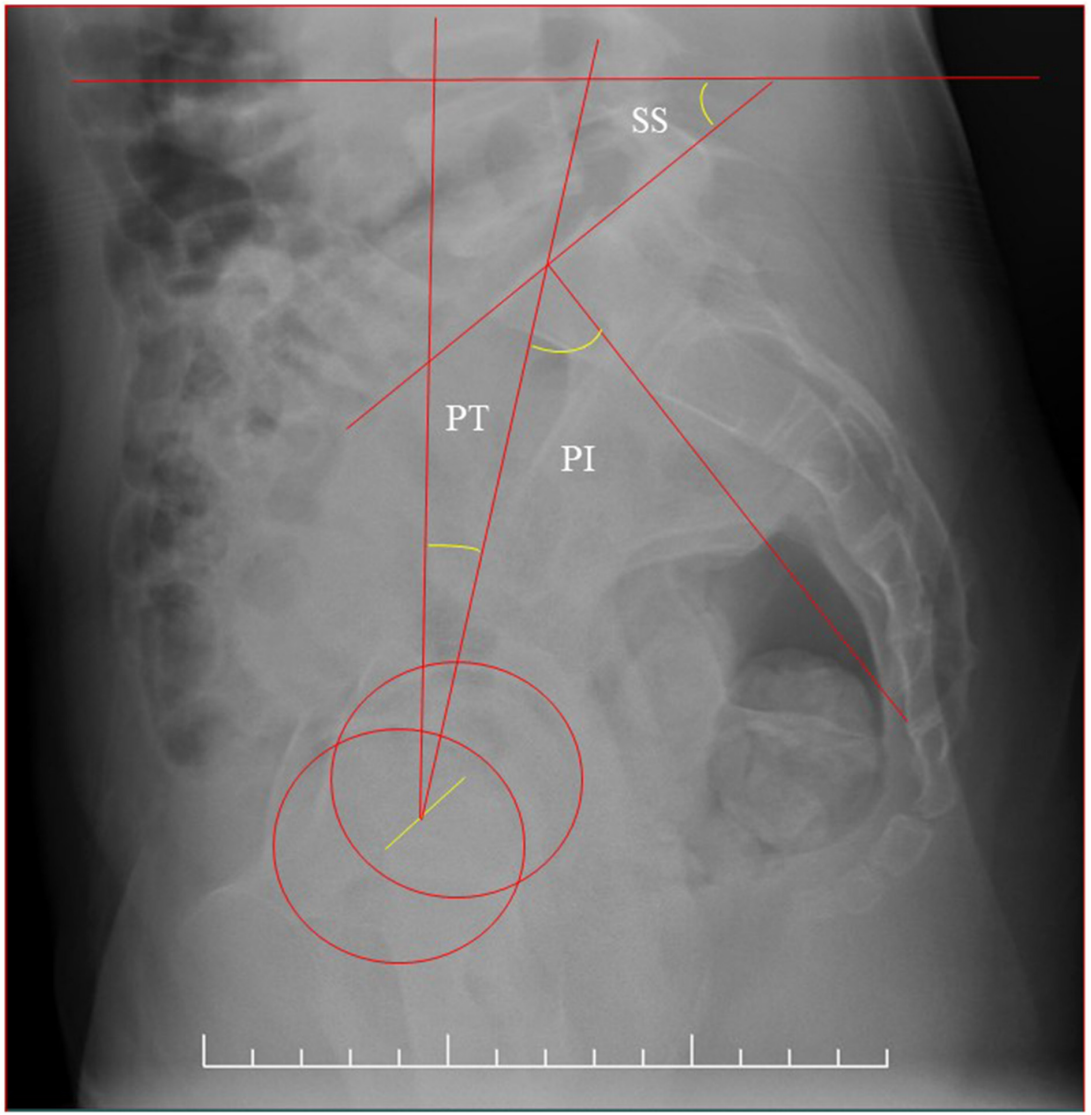
The sagittal balance parameters of SS, PT, PI.

Radiographs were evaluated for the following spine and pelvic measurements (Figures 1, 2). Coronal parameters included thoracic cobb angle (TC), lumber cobb angle (LC), global coronal balance (GCB), apical translation of the major curve (AT), coronal pelvic tilt (CPT) were analyzed. Sagittal parameters included thoracic kyphosis (TK), lumber lordosis (LL), sagittal vertical axis (SVA), pelvic incidence (PI), pelvic tilt (PT), and sacral slope (SS) were analyzed. These pelvic parameters were then compared with previously established data from age-matched normal children using 2-tailed Student *t*-tests, by OpenEpi software program.

### Canonical Correlation Analysis

**Figure 3:**
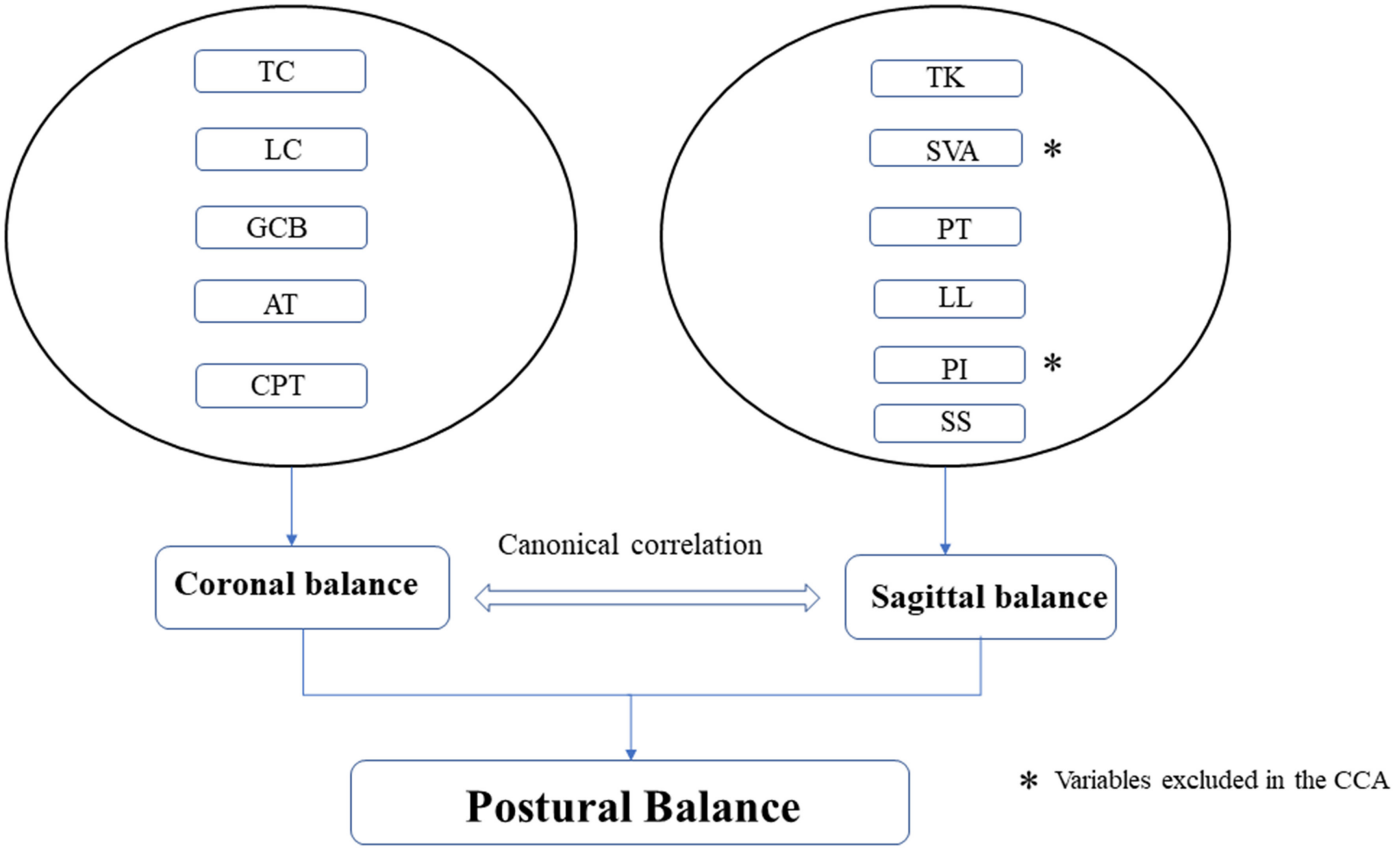
Diagrammatic sketch of the canonical correlation analysis of coronal and sagittal balance.

To analyze the association between the coronal and sagittal balance variables, a canonical correlation analysis was conducted (CCA) (Figure 3). The CCA analysis permits to investigate the association between two groups of variables. In the current study, balance variables of coronal and sagittal were composed of a linear combination of their respective multiple variables. Unlike the Pearson correlation coefficient, which allows for testing two variables, CCA can correlate groups of multi-variants at a time. A detailed tutorial of the CCA can be approached elsewhere (11). The calculation of the power of CCA can be accessed by the online program of Univariate and Multivariate Power Analysis (https://www.albany.edu/~rfh64/power/power.html).

### Statistics

Data analysis was performed with SPSS 25 (IBM Co, USA). The significance level was set at *p* < 0.05 with a 95% confidence interval. Pearson bivariate correlation was evaluated between coronal parameters and SVA. Canonical correlation analyses between the predictors (four sagittal variables) and the predicted (five coronal variables) were conducted. The standardized coefficients were used to account for differences in standard deviations of the variables in the data sets. The variables in each data set that significantly contributed to the canonical correlations were identified using standardized functional coefficients.

## Results

One hundred and three patients patients were recruited (male 15, female 88; aged 12.5 ± 1.5 years) in this study. Patient demographics and radiographic measurements are listed in Table 1. Patients were classified as follows, 47 cases for Lenke I, 8 for Lenke II, 10 for Lenke III, 2 for Lenke IV, 31 for Lenke V, 5 for Lenke VI, according to Lenke's method. The averaged value of TC is 24.8 degrees and LC is 24.1 degrees.

**Table 1:** Demographics, radiographic measurements of adolescent idiopathic scoliosis patients.

	**Min**	**Max**	**Mean ± SD**
Age	10	17	12.51 ± 1.47
LC	5	52	24.13 ± 9.13
TC	5	85	24.81 ± 13.34
AT	0	10.0	2.20 ± 1.55
LPT	0	6.0	1.55 ± 1.40
CB	0	7.2	1.09 ± 0.92
LL	16	73	44.34 ± 11.22
TK	3	63	24.50 ± 11.42
SVA	−8.0	8.0	−0.08 ± 2.69
PT	0	27	8.98 ± 5.53
SS	20	58	38.03 ± 7.45
PI	27	74	47.01 ± 9.06

*Date are presented as means ± SD*.

Compared with the published age-matched normal population, PI, LL, and SS were not statistically different in the normal group. However, PT was significantly different in the normal group (12) (Table 2). SVA was not correlated with any coronal parameter in this study according to Pearson correlation test.

**Table 2:** Sagittal parameters compared with published age-matched normal population.

	**Scoliosis (*n* = 103)**	**Mac-Thiong (*n* = 35)**	***P***
TK	24.5 ± 11.4	38.3 ± 9.8	0.32
LL	44.3 ± 11.2	45.6 ± 12.1	0.51
PI	47.0 ± 9.1	44.6 ± 10.6	0.25
PT	9.0 ± 5.5	4.3 ± 8.1	0.003
SS	38.0 ± 7.4	40.3 ± 8.7	0.22

*Date are presented as means ± SD*.

CCA was performed to evaluate relationship between the coronal parameters (five variables) and sagittal parameters (four variables) synthetic variables, which resulted in four canonical functions. PI was summation of PT and SS, so it was excluded from the sagittal set as variances and supposed to be independent of each other within the same set. The canonical correlation values of these functions (function 1–4) were 0.46, 0.39, 0.27, and 0.04. Since the first canonical function explained 46% of the variance and was statistically significant *F*_(20, 313)_ = 2.44, *p* = 0.001 (Table 3), it was determined that function 1 was noteworthy in test the association between the coronal parameters and sagittal parameters. The standardized canonical function, canonical structure coefficients were presented in Table 4. In the first function of LL, SS, and PT from the predicted dataset and LPT and GCB from the predictor dataset were the main contributors to the synthetic variables of coronal and sagittal parameters, respectively. LL and PT were positively related to LPT and GCB, while inversely related to LC and TC. It can be confirmed that LC, TC, and SS values were all negative in the cohort. The negative sign suggests a positive relationship between them. These variables had a large standardized coefficient (Table 4). In the second function, TC and LC from the predicted dataset and LL and TK from the predictor dataset were the main contributors. TC was positively correlated to LL and TK (Table 4). The estimated power of CCA in our study was about 99.99%.

**Table 3:** The canonical correlation for the functions.

**Correlation**	**Wilks**	***F***	**Num D.F**	**Denom D.F**	***P***
0.46	0.62	2.44	20.00	312.71	0.001
0.39	0.78	2.04	12.00	251.64	0.02
0.27	0.92	1.27	6.00	192.00	0.27
0.04	1.00	/	/	/	/

**Table 4:** The standardized canonical correlation coefficients of coronal and sagittal balance.

	**Function 1**	**Function 2**
Variable in set 1		
LC	−0.27^*^	−0.51^*^
TC	−0.28^*^	1.01
AT	0.05	0.19
LPT	0.61	−0.12^*^
GCB	0.67	0.21
Variable in set 2		
LL	0.56	0.47
TK	0.32	0.54
PT	0.64	−0.27^*^
SS	−0.78^*^	0.28

* *Data means the canonical correlation coefficients are negative in the function*.

## Discussion

As we all know, this is the first study to explore the relationship between coronal balance and sagittal balance in AIS. Balances of coronal and sagittal planes are both of great importance and frequently studied in literature.

We compared our results with normal children described in the literature. With the exception of increased pelvic tilt, sagittal-plane spinopelvic parameters were similar between children with scoliosis and those reported for teenager children without spinal deformity. El-Hawary's study showed pelvic tilt was significantly higher and sacral slope was significantly lower in children younger than 10 years with scoliosis (13). In both El-Hawary's and our studies, pelvic tilt with spinal deformity was significantly higher than normal children. The pelvic incidence was not significantly different between the scoliosis and normal groups. As we know, pelvic incidence is the summation of pelvic tilt and sacral slope. There was a compensatory mechanism to counterbalance the increased pelvic tilt in the scoliosis group. The mean thoracic kyphosis was smaller as compared to normal children in El-Hawary's study, while it was not statistically significant. The author believed that hypokyphosis in AIS were developed mostly during adolescence instead of preadolescence. SVA was −0.08 ± 2.69 cm in our study, while SVA was 2.2 cm in El-Hawary's study. This could be caused by decreased thoracic kyphosis.

Spine surgeons always strive for better curve correction for AIS in order to help them acquire better life quality. Several authors have investigated the relationship between HRQOL and radiographic parameters. Both coronal curve location and extent were not significantly predictive of life quality in Acaroglu's study (14). Another study showed that coronal imbalance <4 cm does not influence quality of life in scoliosis (15). There was also study advocated that sagittal balance was vital for life quality. For example, SVA and truncal inclination were correlated with HRQOL and Oswestry Disability Index in Lafage's study (10).

Currently, the disability of adults with spinal deformity appears to be rather a concern of the sagittal plane. Given that coronal plane is correlated to sagittal plane in AIS, the author suggests not to omit the potential influence of coronal plane on life quality. One of the more likely indirect effects is the rise in coronal imbalance as life quality gets worse as a result of impaired sagittal balance.

When considering how to treat AIS, both coronal balance and sagittal balance should be taken into account, but which one of two balances is more important is still unanswered. Our results showed positive correlation between coronal variables and sagittal variables and suggested the importance of considering both coronal balance and sagittal balance for a comprehensive balance evaluation. The strong relationship can guide correction of the spinal deformity and should be considered for guiding the spinal fusion such as selection of lower instrumented level as well as the conservative methods such as the application of orthosis and Schroth exercise in AIS.

We examined five coronal parameters and six sagittal parameters in this study. If Pearson bivariate correlation was conducted for each pair of parameters, there would be 5 × 6 = 30 coefficients. For example, thoracic cobb angle was positively correlated to lumber lordosis (*P* = 0.001, *r* = 0.32, values were not listed in the results to avoid redundancy). To list pairwise correlations was technically achievable but helpless for evaluating the overall association between two sets of variances. That's why we chose canonical correlation analysis for multivariance comparison.

This study was dedicated to explore the correlation between the sagittal alignment and coronal status of the spine. Two major limitations exist in this work. First, although two main planes of the spine were involved in the study, the axial rotation was not studied and the correlation between axial rotation and coronal/sagittal balance could not be assessed. In order to measure axial rotation precisely, EOS was recommended instead of 3D CT reconstruction due to much lower radiation exposure (16). However, EOS currently is not universally available. Second, many parameters were used in this study to confirm a correlation between coronal and sagittal balance. Unfortunately, we still don't have one specific parameter that can represent the general status of coronal or sagittal balance.

More work is needed to elucidate the reasons of the correlation rather than only identifying the correlation. In another study (17), we had done some research in estimating the relationship between coronal balance and static baropodometry in AIS patients, and we found that coronal balance is correlated with planter pressure distribution. Additionally, in order to put the current study into practical use, further studies are definitely needed. One possible solution is to design an index of overall balance which is in linear correlations with the balance parameters involved in our study and such index would facilitate surgeon actions in the near future. We will pay more attention to the relationship between the curve type or size and postural balance, this may provide more practical application to clinical management in AIS surgery. And then care should be taken of overall balance instead of just coronal or sagittal balance for higher life quality.

## Conclusions

A basic goal in the treatment of spinal deformity is to achieve proper alignment. To achieve this purpose, the surgeon must pay attention to global spinal balance.

The following main points can be concluded from the data of this investigation. Children with AIS showed signs of increased pelvic tilt and decreased TK. In AIS, coronal balance is correlated to sagittal balance.

We believe coronal balance and sagittal balance are equally important for decision-making when dealing with AIS.

## Data Availability Statement

The raw data supporting the conclusions of this article will be made available by the authors, without undue reservation, to any qualified researcher.

## Ethics Statement

The studies involving human participants were reviewed and approved by The Institutional Review Board of Shanghai Children's Hospital. Written informed consent to participate in this study was provided by the participants' legal guardian/next of kin.

## Author Contributions

YL and ZL conceived and designed the study. QM, LW, YW, and MC collected the data. QM, SW, ZL, and YL analyzed and interpreted the patient data. QM and YL wrote the paper. QM, YL, and ZL revised the paper. All authors read and approved the final draft. All authors approved the final manuscript as submitted and agree to be accountable for all aspects of the work.

### Conflict of Interest

The authors declare that the research was conducted in the absence of any commercial or financial relationships that could be construed as a potential conflict of interest.
